# Synergistic effect of acupuncture and mirror therapy on post-stroke upper limb dysfunction: a study protocol for a randomized controlled trial

**DOI:** 10.1186/s13063-018-2585-8

**Published:** 2018-05-31

**Authors:** Ying Xu, Shufang Lin, Cai Jiang, Xiaoqian Ye, Jing Tao, Schupp Wilfried, Alex W. K. Wong, Lidian Chen, Shanli Yang

**Affiliations:** 1 Rehabilitation Medicine College, Fujian University of Traditional Chinese Medicine, Fuzhou, 350122 China; 2 Rehabilitation Hospital Affiliated to Fujian University of TCM, Fuzhou, 350003 China; 3 Fujian University of Traditional Chinese Medicine, Fuzhou, 350122 China; 4 M&i-Fachklinik Herzogenaurach, 91074 Herzogenaurach, Germany; 5 Program in Occupational Therapy & Department of Neurology, Washington University School of Medicine, St. Louis, MO 63108 USA

**Keywords:** Acupuncture, mirror therapy, rehabilitation, stroke, upper limb dysfunction

## Abstract

**Background:**

Upper limb dysfunction is common after stroke, posing an important challenge for post-stroke rehabilitation. The clinical efficacy of acupuncture for the recovery of post-stroke upper limb function has been previously demonstrated. Mirror therapy (MT) has also been found to be effective. However, the effects of acupuncture and MT have not been systematically compared. This trial aims to elucidate the synergistic effects of acupuncture and MT on upper limb dysfunction after stroke.

**Methods:**

A 2 × 2 factorial randomized controlled trial will be conducted at the rehabilitation hospitals affiliated with Fujian University of Traditional Chinese Medicine. A total of 136 eligible subjects will be randomly divided into acupuncture treatment (AT), MT, combined treatment, and control groups in a 1:1:1:1 ratio. All subjects will receive conventional treatment. The interventions will be performed 5 days per week for 4 weeks. AT, MT, and combined treatment will be performed for 30 min per day (combined treatment: AT 15 min + MT 15 min). The primary outcomes in this study will be the mean change in scores on both the FMA and WMFT from baseline to 4 weeks intervention and at 12 weeks follow-up between the two groups and within groups. The secondary outcomes are the mean change in the scores on the Visual Analogue Scale, Stroke Impact Scale, and modified Barthel index. Medical abstraction of adverse events will be assessed at each visit.

**Discussion:**

The results of this trial will demonstrate the synergistic effect of acupuncture and MT on upper limb motor dysfunction after stroke. In addition, whether AT and MT, either combined or alone, are more effective than the conventional treatment in the management of post-stroke upper limb dysfunction will also be determined.

**Trial registration:**

Chinese Clinical Trial Registry: ChiCTR-IOR-17011118. Registered on April 11, 2017. Version number: 01.2016.09.1.

**Electronic supplementary material:**

The online version of this article (10.1186/s13063-018-2585-8) contains supplementary material, which is available to authorized users.

## Background

Upper limb dysfunction is a common dysfunction after stroke. Of 65–80% of patients with upper limb dysfunction, 37% have varying degrees of difficulty with upper limb fine motor control [1]. Furthermore, approximately 50% are unable to complete activities of daily living (ADL) independently 3 months after stroke onset [2]. Post-stroke upper limb dysfunctions, including coordination and execution problems of the arms, palms, and fingers, cause limitations in eating, dressing, showering, and other daily activities [3], increasing the financial burden on families and society. Therefore, improving upper limb dysfunction is a significant priority for stroke rehabilitation so that patients can improve their abilities to perform ADL and optimize their quality of life.

A variety of rehabilitation treatments are available for upper limb dysfunction after stroke, including upper limb motor training, adaptive equipment or aids, and traditional Chinese medicine (TCM). However, management of upper limb dysfunction after stroke is complicated, and a single treatment is not adequate. Thus, comprehensive multidisciplinary effective measures may significantly improve the level of rehabilitation.

Currently, both acupuncture and mirror therapy (MT) are widely used for stroke rehabilitation, with MT being gradually applied by clinical researchers as a new treatment for post-stroke limb rehabilitation [4–6], particularly for severe upper limb and pain rehabilitation [7]. One study revealed that, after 1 month of MT, the functional independence measure score increased from 52 ± 17.16 to 93.18 ± 22.07, the action research arm test score increased from 15.90 ± 22.41 to 47.64 ± 15.19, and the motricity index increased from 39.27 ± 27.33 to 76 ± 21.78. Therefore, compared to conventional treatment, MT is a promising and simple method of improving motor recovery of the upper limbs of subacute stroke patients [8].

Acupuncture originated in ancient China approximately 2500 years ago as an important component of TCM. It has been used for thousands of years in several Asian countries to treat a variety of diseases. Acupuncture is simple and effective and has no adverse drug reactions. It has become a popular topic in the field of rehabilitation medicine in China. However, whether acupuncture promotes recovery from stroke remains controversial. Several studies have demonstrated that acupuncture treatment (AT) may promote the recovery of upper limb dysfunction after stroke [9–11]. One study involving a post-hoc analysis showed that the electrical stimulation group had greater improvements in hand grip (*P* = 0.015) and pinch strength (*P* = 0.007) than the control group after 4 weeks [12]. However, a systematic review showed that the methodologic quality of the included trials was inadequate and that the results may not be reliable because of substantial heterogeneity [13].

Acupuncture is a state-of-the-art TCM treatment widely utilized in China. The advantages of AT are its simplicity, convenience, affordability, and efficacy. The use of AT for the treatment of upper limb dysfunction associated with stroke is in alignment with China’s healthcare development priorities. Furthermore, MT is an important component of modern Western rehabilitation. The combination of AT and MT not only embodies the combination of Chinese and Western medicine but also reflects the integration of modern and traditional rehabilitation. If the rehabilitation program is proven to be effective, then its clinical effectiveness will be important for patients requiring rehabilitation. In addition, it is unknown whether the outcomes of combined AT and MT are superior to those of MT alone because very few studies have compared the two therapies.

Therefore, we designed a 2 × 2 factorial randomized controlled trial to explore the clinical efficacy of AT combined with MT to improve the upper limb motor function of stroke patients. We hypothesized that the synergistic effect of both therapies is greater than the sum of the individual effects of each intervention if they are used separately. AT or MT significantly improved upper limb function outcomes to a similar extent compared with the control group.

During this trial, our first aim will be to assess the synergistic effects of AT and MT on the recovery of upper limb function after stroke. Our second aim will be to determine whether AT and MT, alone or in combination, are more effective than conventional treatment for the rehabilitation of upper limb dysfunction after stroke.

## Methods

### Study design and setting

**Fig. 1:**
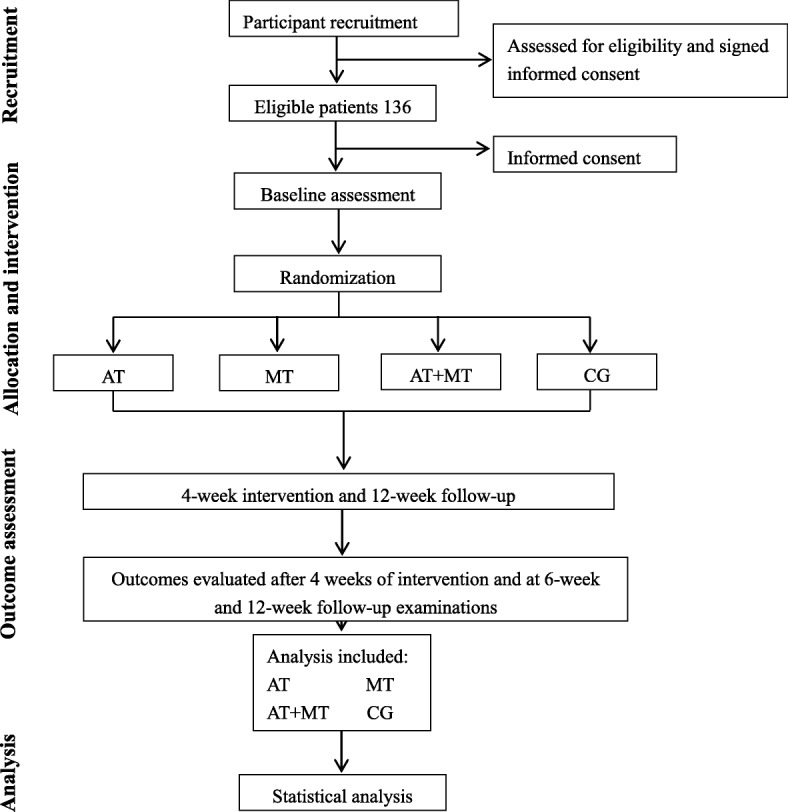
Flow diagram of study design

This trial will use a 2 × 2 factorial randomized controlled study to explore the clinical efficacy of AT combined with MT for improving the upper limb motor function of stroke patients. It will be performed in the Rehabilitation Hospitals Affiliated with Fujian University of Traditional Chinese Medicine (FJTCM). A total of 136 eligible subjects will be randomly divided into four groups, namely (1) AT group, (2) MT group, (3) combined treatment group (AT + MT), and (4) control group, in a 1:1:1:1 ratio. All subjects will receive conventional treatment, including health education and conventional medical therapy. The interventions will be performed 5 days per week for 4 weeks. AT, MT, and combined treatment (AT 15 min + MT 15 min) will be performed for 30 min per day. Therapists with more than 5 years of clinical experience will perform the interventions. A 4-week intervention and 12-week follow-up period will be conducted. Outcome measurements include the Fugl–Meyer Assessment for Upper Extremity (FMA-UE), Wolf Motor Function Test (WMFT), Visual Analogue Scale (VAS), Stroke Impact Scale (SIS), Modified Barthel Index (MBI), and adverse events. The flow diagram for this trial is presented in Fig. 1. The undertaking unit of this project (the Rehabilitation Hospitals Affiliated with FJTCM) and the ethics committees are responsible for making protocol decisions and for communicating important protocol modifications. In addition, the project undertaking unit is responsible for coordinating the work of all departments (e.g., trial registries, researcher training, informed consent of the participants, data management). If there are important protocol modifications, the project undertaking unit will notify relevant parties to convene a coordination meeting.

### Sample size

Sample size was calculated based on the improvement in FMA-UE scores. Based on similar previous reports, the mean difference and SD were 42.86 ± 3.74 and 41.68 ± 3.44 for AT and conventional therapy, respectively [14]. We expect an effect size of at least 0.3 for outcomes after intervention. A sample size of 112 participants is required to sufficiently detect a target effect size with a type 1 error of 5% (α = 0.05) and 80% power (β = 0.20) by using Gpower 3.1.9.2 software. Considering a 20% attrition rate, a total of 136 participants are necessary, with 34 participants in each group.

### Participants and recruitment

**Fig. 2:**
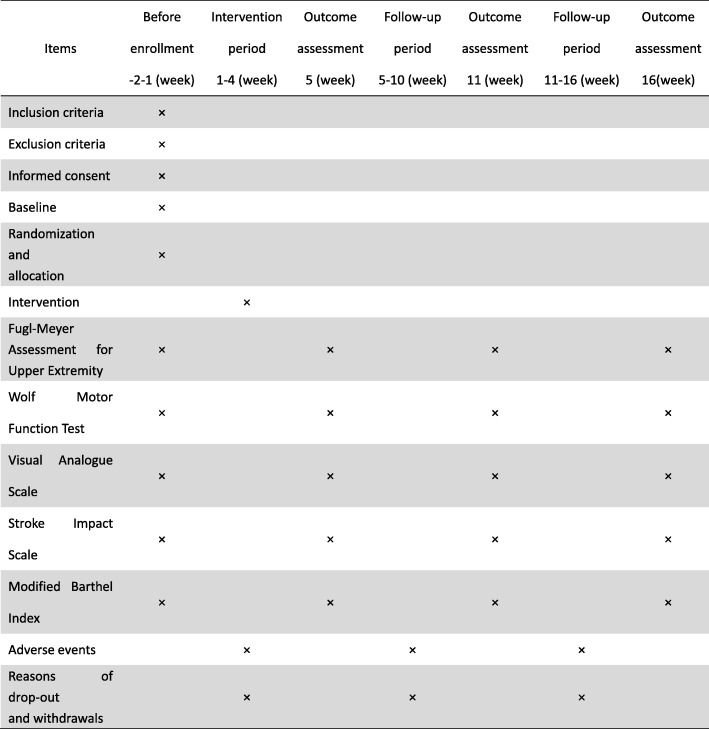
A Standard Protocol Items: Recommendations for Interventional Trials (SPIRIT) Figure

We will recruit 136 eligible patients with upper limb motor dysfunction caused by stroke from the rehabilitation hospitals affiliated to FJTCM. Eligible patients must meet the inclusion and exclusion criteria. A Standard Protocol Items: Recommendations for Interventional Trials (SPIRIT) Figure is shown in Fig. 2. Information about this trial will be available through the hospital website, and leaflets will be distributed to inpatients and outpatients at multiple locations in the hospital. Interested patients can contact the project leader through their therapists, among others, by telephone and e-mail. If an applicant meets the study criteria, then they will be invited to participate.

### Inclusion criteria

Eligible patients are those (1) diagnosed with stroke and confirmed by computed tomography or magnetic resonance imaging; (2) first-ever clinical stroke occurred ≤ 6 months previously; (3) FMA-UE score of ≤ 33 points; (4) modified Ashworth score of grade II or lower; (5) aged between 18 and 80 years; (6) stable vital sign and consciousness; and (7) provided written informed consent to participate.

### Exclusion criteria

Criteria for exclusion are (1) upper limb dysfunction caused by brain tumor, brain trauma, and other diseases; (2) Montreal cognitive assessment score ≤ 26 points (if the education period is less than 12 years, then add 1 point to the score); (3) bone disease or joint damage causing limb deformities; (4) serious disease in the heart, liver, kidney, hematopoietic system, or endocrine system; and (5) participation in other clinical trials that may affect the results of this study.

### Withdrawal criteria and management

Participants will be allowed, or required, to withdraw from the trial based on (1) a major protocol violation; (2) development of a serious disease preventing continuation in the trial; (3) adverse events related to AT or MT; and (4) request to be withdrawn from the trial.

### Randomization and allocation

The randomization sequence will be created using SAS 9.1 by an independent statistician who works in the Evidence-Based Medicine Center of FJTCM. The allocation of participants will be concealed using envelopes that are sequentially numbered, opaque, and sealed. Competent doctors will work in strict accordance with the inclusion and exclusion criteria to determine whether the subjects can participate in the study. The investigator will then number the cases in chronological order. Screeners will assign the eligible number and opaque envelopes (containing random numbers and allocation and intervention information) to the therapists. The treating therapists will be notified of the allocation and make arrangements directly with the patient to administer the allocated treatment. Eligible participants will be informed of their group allocation by the project manager after their baseline information has been assessed. Researchers who determined the random numbers and performed the allocation will not participate in subject screening.

### Blinding

A single-blind method will be used. The outcome assessors, data manager, and statistics analyzer will evaluate the results of the study without being aware of subject allocation. The results of the assessment will be kept confidential to the investigator, thereby improving the accuracy of the test indicators, ensuring the effectiveness of the interventions, and assuring the objectivity and reliability of the evaluation. Certain staff will supervise the blind implementation process. The blind code will be disclosed after completion of the statistical analysis.

### Treatment allocation

The interventions will be performed 5 days per week for 4 weeks. AT and MT will be performed for 30 min per day, and combined treatment (AT 15 min + MT 15 min) will be performed for 30 min per day. During the study period, precise documentation of treatments, medication, and lifestyle-related influencing factors will be recorded in the case report forms (CRFs). All of the employees participating in this trial will receive standardized training regarding the study protocol, treatment methods, and assessments. The groups and intervention allocation are listed in Table 1.

**Table 1:** Groups and intervention allocation

Groups	Interventions
Acupuncture treatment (AT) group	Conventional treatment + AT
Mirror therapy (MT) group	Conventional treatment + MT
Combination (AT + MT) group	Conventional treatment + AT + MT
Control group	Conventional treatment

### Conventional treatment

#### Basic treatment

All patients will receive conventional treatment provided by the rehabilitation hospitals affiliated to FJTCM. According to the *Chinese prevention and treatment guidelines of cerebrovascular disease* [15], the doctors will develop a basic treatment program based on the specific characteristics of individual patients. This means that patients will receive treatments deemed necessary by the doctors, such as conventional medical treatment, physical therapy, occupational therapy, and speech-language therapy, while receiving intervention. There are no restrictions for the aforementioned situations. We will track the types of interventions provided and precisely document the therapy treatments and medication.

#### Health education

We will use health education manuals to perform health education. The main contents of the manuals include (1) the severity of stroke to encourage people to focus enough attention and perform positive preventive measures actively; (2) the main risk and predisposing factors for stroke and secondary stroke prevention; and (3) management of and rehabilitation for stroke. For example, the manual describes when the best time to see the doctor is and how to cooperate with medical staff during treatment and rehabilitation.

### Mirror therapy

During initial treatment, the definition and content of MT will be explained to patients by an occupational therapist with more than 5 years of clinical experience. Patients will be instructed to sit close to a table on which a 35 × 35 cm mirror is vertically placed and then to put the upper limbs on the table. The contralateral upper limb will be positioned toward the reflective surface of the mirror, and the ipsilateral upper limb will be positioned on the opposite side of the mirror. Finally, patients will be required to observe the movement of the contralateral upper limbs in the mirror and to imagine the affected side in motion. Then, they will be required to perform the movement of the affected side as consistently as possible (if patients cannot complete the movement, then the experienced therapist will assist). Patients will need to complete six movements, namely shoulder flexion, elbow flexion and extension, forearm pronation, wrist flexion and extension, finger stretching, and thumb outreach. Patients will be asked to achieve a maximum range of motion for 30 min per day for 5 days per week for 4 weeks.

### AT protocol

Participants will be treated using five acupuncture points on the hemiplegia side (Jianyu [LI15], Quchi [LI11], Shou sanli [LI10], Hegu [LI4], and Waiguan [TE5]) by an acupuncturist with more than 5 years of clinical experience. AT will be performed with patients in the supine position. Points will be sterilized with alcohol, and the target position will then be fixed by the acupuncturist’s left hand. The acupuncturist’s right hand will insert the thin, disposable acupuncture needles (Huatuo, single use, 0.30 × 40 mm; Suzhou HuaTuo Medical Instruments, Suzhou, China).

The angle of insertion will be 10°–20° (between the needle and skin), and the needle depth will be approximately 1.0–1.5 B-cun. Following insertion, stimulation of the acupuncture point will be performed with bidirectional rotation of the needle sleeve at approximately 18–300 per min to achieve the sensation known as *Deqi*, which is commonly described as a glowing feeling.

The needle will be maintained in place for 30 min. A sterilized, dry cotton ball will be gently pressed against the point to prevent bleeding when each needle is withdrawn. The entire course of treatment will continue for 30 min per day for 5 days per week for 4 weeks.

### AT combined with MT

In the combined treatment group, patients will receive AT (15 min) and MT (15 min). However, the two treatments will be performed separately, yet the order of the two treatment methods will not be limited. Each treatment will last 15 min, for a total of 30 min per session.

### Follow-up

All participants will participate in a 12-week unsupervised follow-up period immediately after the end of intervention. Telephone follow-up will be performed once every 4 weeks, and records of the medications and rehabilitation therapies will be kept. During the 6- and 12-week follow-up periods (10 weeks and 16 weeks after intervention), participants will be referred for clinical evaluation to assess their functional status, including upper limb function, quality of life, and ADL.

### Outcome assessment

The study period is 16 weeks. Outcomes will be assessed at baseline, after 4 weeks of intervention, at 6-week follow-up, and at 12-week follow-up. All outcomes will be measured by several experienced assessors who are blinded to the randomization group after the baseline visit for evaluation. All assessors will be trained to administer these assessments.

### Primary outcomes

The primary outcomes in this study are the mean change in the scores on both the FMA and WMFT from baseline to 4 weeks intervention and 12 weeks follow-up between the two groups and within groups.

The FMA is widely used for motor function assessment and can reflect the functional level of stroke subjects. Even during the early stage of functional recovery, it can distinguish the level of motor function, particularly for those who have low responsiveness. The upper extremity has a maximum score of 66 points divided into three parts, namely shoulder–arm (36 points), wrist–hand (24 points), and coordination (6 points). The FMA-UE is the most common scale for evaluating upper limb motor function after stroke. It is often used as a gold standard for testing the validity of other scales as well [16, 17]. The FMA-UE has excellent reliability and validity and is sensitive enough for clinical and research practice [18].

The WMFT is the preferred scale for assessing mandatory exercise therapy for improving upper limb functions. It can either assess the quality of the patient’s completion of each activity or determine the time required for the patient to do so. The WMFT has good reliability and validity and high internal consistency [19].

### Secondary outcomes

The mean change in the scores on the VAS, SIS, and MBI after 4 weeks intervention are considered secondary outcomes.

In this study, the VAS will be used to evaluate the highest overall pain level of the upper limb on the affected side during the week before the assessment. The basic method is to use a 10-cm horizontal line (one marked with 10 scales, with endpoints “0” and “10”), which allows patients to indicate the degree of pain. Zero indicates no pain and 10 indicates the most intense pain. In clinical practice, the VAS is considered the most sensitive and reliable method for measuring pain [20].

SIS is mainly used to evaluate the impact of stroke on the health and quality of life of patients [21]. The scale involves items measuring dysfunction caused by stroke and the impact on the patient’s life, with scores ranging from 0 to 100, and assesses the patients’ overall perceptions of recovery after stroke. The scale includes eight domains (strength, hand function, ADL/instrumental ADL, mobile ability, communication, mood, memory and thinking, and participation) and has a total of 59 items.

MBI is the most commonly used internationally recognized evaluation tool for testing the ability to perform ADL [22]. This scale includes items related to bowel and bladder continence, grooming, toileting, feeding, transfer, walking, dressing, bathing, and going up and down stairs. A normal score is 100, and lower scores indicate greater dependency.

### Safety assessments

Acupuncture may cause discomfort or bruising at the sites of needle insertion, nausea, or feeling faint after each treatment. No adverse reactions caused by MT have been reported to date.

During the course of the study, any accidental injury and sudden illness will be recorded. The degree of symptoms, time of occurrence, duration, and treatment will be documented in the observation table. All adverse events reported should be analyzed regardless of the investigators’ assessments of causality. Serious adverse events will be reported to the ethics committee immediately.

### Data collection and management

The data will be entered into a carefully designed electronic CRF. A dependent research assistant (RA) will be in charge of quality control during the data collection process. The project leader will be responsible for the initial data cleansing, identification, coding, and conversion to the appropriate data analysis format. In addition, an independent data and safety monitoring board will be established to monitor the safety of the trial. It will comprise two professional therapists who are experienced in the rehabilitation of limb movement dysfunction after stroke and who are not directly involved in this study. The data and safety monitoring board is independent and will be responsible for the effectiveness and management of data and reports of adverse events.

Demographic information, medical history and medication, lesions, affected side, handedness, cognitive function, time since stroke, stroke type, rehabilitation history (treatment until the beginning of the study), details of current and upper limb dysfunction treatment received within the previous 3 months, and concomitant medications will be recorded. Data will be obtained through direct questions to patients or a relative or proxy and from medical records. The outcome measure will be performed by an experienced assessor after intervention and follow-up. All forms must be dated and signed by the responsible investigator or one of the authorized staff members.

The project manager will have access to the data, and all data is treated with utmost confidentiality and made anonymous to anyone outside the study. All researchers in this study will receive anonymous copies of all data in order to promote dissemination of study results. The result of our study will be published in a peer-reviewed journal.

### Statistical analysis

All allocated subjects will be analyzed using the available data (i.e., on the basis of the intention-to-treat). A multiple imputation method will be used to complete missing data. Continuous variables will be expressed as mean (SD) for normal distribution or median for non-normal distribution, and categorical variables will be expressed as frequencies or percentages. A significance level of 95% (two-sided alpha *P* < 0.05) will be used.

After examining the data for normality (Kolmogorov–Smirnov test), the outcome measures will be analyzed using analysis of variance (assuming normal distribution) or Kruskal–Wallis (non-parametric). Demographic characteristics and other baseline values will be described using descriptive statistics for each group. Intragroup comparisons (changes in the FMA-UE, WMFT, VAS, SIS, and MBI scores before and after treatment) will be performed using the paired *t* test. The statistical significance of the observed differences between the groups will be assessed using 2 × 2 factorial analysis of variance to test the main effect and interaction effects of the two interventions (AT and MT). A statistician blinded to study groups will conduct statistical analysis.

### Quality control

- Quality control of informed consent: We will randomly select 20% of the participants to determine their knowledge about the study and awareness of signed informed consent. If they are knowledgeable, then this procedure will pass. Otherwise, the trained RA will need to explain the study again to all participants and randomly select 40% of the participants to determine their knowledge. If they are knowledgeable, then the procedure will pass; otherwise, the trained RA will explain again.
- Quality control of participant screening: Participant screening must be performed according to the inclusion and exclusion criteria. If participants meet all the criteria, then this procedure will pass. On the contrary, participants who do not meet the criteria will be excluded, and the number and reasons will be recorded.
- Quality control of intervention: The project manager will supervise the AT therapist and MT therapist who will treat the participants, and the MT therapist will supervise the intervention process of the participants. At the same time, an independent inspector will review the trial process, including the treatment of the subjects, the procedures of the therapist, and the registration of adverse events.
- Quality control of statistical analysis: Missing data are not allowed in report forms, particularly for the security index, which must be recorded clearly. Basic demographic data, such as sex, date of birth, and date of inclusion, among others, should be recorded to distinguish between missing data. If the missing data are left out after checking the original data, then we need to complete the missing parts. If the missing data are the security index, then we will not record them in the original data for security; rechecking of data will be necessary. The security index will be removed if it affects the results.
- Quality control of data management: The project manager will preserve and check all file lists. If there are missing data, then related personnel will be required to complete what is missing. Then, we will randomly select 10% of the CRF to check whether there any data errors. All data will be independently double-entered into a computer. The CRF questionnaire will be submitted to the investigator via the clinical examiner for data auditing, and the investigator should reply as soon as possible thereafter.

## Discussion

The incidence of upper limb dysfunction caused by stroke can reach up to 66% [23]. Every year, approximately 70–80% of new patients lose their ability to work to varying degrees and cannot take care of themselves, placing a heavy burden on patients, families, and society [24]. Upper limb motor dysfunction is a great challenge during stroke rehabilitation.

Studies have confirmed that direct current stimulation, strength training, robot-assisted training, virtual reality therapy, constraint-induced movement therapy, and other rehabilitation therapies could achieve different degrees of improvement about upper limb function after stroke [25–27]. Furthermore, AT and MT seem to be beneficial for the recovery of upper limb dysfunction after stroke [28, 29].

The effect of AT is mainly achieved by stimulating acupuncture points called “acupoints,” which are specific parts of the body used to gather Qi and blood. Acupoints are not only the external reactions of internal diseases in the body but also points for the treatment of disease. The specific effect of AT on diseases is mainly elicited through activating Qi and blood, as well as balancing Yin and Yang [30]. AT can adjust the upper limb meridians and help alleviate tendon stagnation, which would eventually help the normalization of the upper limb function.

MT is based on the mirror neuron system. It uses the plane mirror imaging principle for the contralateral activity images projected on the affected side. It also combines optical illusions, visual feedback, and virtual reality [31]. One study has further indicated that MT can promote the recovery of limb function after stroke, thereby improving the ability to perform ADL [32].

However, the effects of AT and MT have not been systematically compared, and the possibility of a synergistic effect of their combination has not been evaluated. Therefore, we propose a rigorous, randomized, controlled clinical trial to demonstrate that the two treatment methods applied alone or in combination are more effective than the conventional treatment. In this study, we will systematically evaluate the effects of different treatments on upper limb motor dysfunction after stroke and assess the upper limb motor function and daily activity. We will also judge the long-term stability of improvements during a 12-week follow-up examination after intervention.

The major limitation of this protocol is its non-double-blind design. However, the outcome assessors and statistical analysts will be blind to the intervention to decrease possible bias and ensure the quality of this trial. This study also lacks long-term follow-up observations and assessments. The 4-week intervention cycle reflects its application in real-world clinical practice and is sufficient to test whether the rehabilitation program is effective in the short term.

In conclusion, the results of this study are expected to elucidate the synergistic effects of AT and MT on upper limb dysfunction after stroke. Furthermore, the results are expected to confirm whether AT and MT, either combined or alone, are more effective than conventional treatment for the management of post-stroke upper limb dysfunction (Additional file 1).

### Trial status

Ongoing recruitment.

## Additional file

Additional file 1: SPIRIT 2013 checklist. (DOC 122 kb)

## Abbreviations

ADL: activities of daily living
AT: acupuncture treatment
CRFs: case report forms
FJTCM: Fujian University of Traditional Chinese Medicine
FMA-UE: Fugl–Meyer Assessment for Upper Extremity
MBI: Modified Barthel index
MT: mirror therapy
RA: research assistant
SIS: Stroke Impact Scale
TCM: traditional Chinese medicine
VAS: visual analogue scale
WMFT: Wolf Motor Function Test
